# Fabrication of a Novel Electrochemical Sensor Based on Carbon Cloth Matrix Functionalized with MoO_3_ and 2D-MoS_2_ Layers for Riboflavin Determination

**DOI:** 10.3390/s21041371

**Published:** 2021-02-16

**Authors:** Rayhane Zribi, Antonino Foti, Maria Grazia Donato, Pietro Giuseppe Gucciardi, Giovanni Neri

**Affiliations:** 1 Department of Engineering, University of Messina, C.da Di Dio, I-98166 Messina, Italy; zribi.rayhane@gmail.com; 2 CNR IPCF Istituto per i Processi Chimico-Fisici, viale F. Stagno D’Alcontres 37, I-98156 Messina, Italy; foti@ipcf.cnr.it (A.F.); donato@ipcf.cnr.it (M.G.D.); gucciardi@ipcf.cnr.it (P.G.G.)

**Keywords:** molybdenum disulphide nanosheets, molybdenum oxide, electrochemical sensors, riboflavine sensing

## Abstract

The preparation and characterization of a hybrid composite, based on carbon cloth (CC) matrix functionalized with two-dimensional (2D) MoS_2_ flakes and MoO_3_, and its use for developing an electrochemical sensor for the determination of riboflavin (RF) is here reported. The 2D-MoS_2_-MoO_3_CC composite was prepared by depositing 2D-MoS_2_ nanosheets, obtained by liquid phase exfoliation (LPE), on the surface of a carbon cloth fiber network, previously functionalized with a layer of molybdenum oxide (α-MoO_3_) by radio-frequency magnetron reactive sputtering technique. The 2D-MoS_2_-MoO_3_CC composite was characterized by scanning electron microscopy and energy dispersive X-ray analysis (SEM-EDX), and Raman spectroscopy. An electrochemical sensor has been then fabricated by fixing a slice of the 2D-MoS_2_-MoO_3_CC composite on the working electrode of a screen-printed carbon electrode (SPCE). The 2D-MoS_2_-MoO_3_-CC/SPCE sensor display good electrochemical characteristics which have been exploited, for the first time, in the electroanalytical determination of riboflavin (RF). The sensitivity to RF, equal to 0.67 µA mM^−1^ in the linear range from 2 to 40 µM, and a limit of detection (LOD) of 1.5 µM at S/N = 3, demonstrate the promising characteristics of the proposed 2D-MoS_2_-MoO_3_-CC/SPCE electrochemical sensor for the determination of riboflavin.

## 1. Introduction

The discovery of graphene has been a revolution in the field of nanomaterials because of its inherent two-dimensionality [1]. This characteristic has given the origin at a very impressive research for using graphene in many devices. For example, two-dimensional electron confinement of ultra-thin 2D graphene has improved the electrical properties compared to other nanomaterials [2]. With the time, a lot of works mentioned a major disadvantage in using graphene, essentially the lack of band-gap.

Nowadays, research is focused on other 2D nanomaterials, such as transition metal disulphides (TMDs), due to their amazing properties. For example, they possess sizable band-gaps around 1–2 eV, promising interesting applications in chemical sensors. Molybdenum disulphide (MoS_2_) is one of the most interesting TMDs nanomaterials. It has an indirect band-gap of 1.29 eV [3] that turns to semiconductor with a direct band-gap of 1.9 eV [4] going from bulk to a monolayer, offering an important charge carrier mobility. MoS_2_ nanosheets have been fabricated via liquid phase exfoliation (LPE) by our groups and used for the detection of different biomolecules [5,6,7,8].

In a previous paper [9], we also investigated the electrochemical properties of MoO_3_, a semiconductor oxide with a band gap of 3.2 eV which presents three crystalline structures among of which the orthorhombic (α-MoO_3_) phase is the most thermodynamically stable. In order to increase its poor conductivity and to improve the electrochemical characteristics, a layer of α-MoO_3_ was grown on flexible conductive carbon cloth (CC). It was demonstrated that the high electron mobility and the large surface area provided by the carbon fibers increased the capacities of the sensor in terms of peak current and sensitivity compared to bare screen-printed carbon electrode (SPCE) [9]. The main advantage of using the CC was its capability to favor the electron transfer between the electrode surface and the analyte. These enhanced electroanalytical properties were demonstrated in the sensing of dopamine [9].

Considering the promising properties offered by 2D-MoS_2_ and α-MoO_3_, the combination of these two materials appears to be very interesting in the fabrication of an electrochemical sensors. These systems have been reported to improve the electrical properties, enhancing the supercapacitor performance, the storage properties and hydrogen evolution [10,11,12,13,14]. Hybrid MoO_3_/MoS_2_ composites reveal more satisfying electrochemical properties than pure MoO_3_ and MoS_2_ [10]. The dispersion of single [11,12,13], or hybrid layers [14] on the surface of carbon cloths has been reported for obtaining better characteristics.

Little has been instead reported on their use for molecular sensing. In this article, we propose an electrochemical sensor for riboflavin (RF) detection using carbon cloth functionalized with MoO_3_ and decorated with 2D-MoS_2_ nanosheets. Riboflavin is a water soluble vitamin of B group and is an important constituent of flavoproteins, and plays a vital role in the enzyme reactions in the human body [15] as well as for animals [16], that need a riboflavin supply to maintain a stable balance in their bodies. Supplemental RF intake appears to have a protective effect on various medical conditions such as sepsis, ischemia and at the same time it also helps to reduce the risk of certain forms of cancer in humans and has anti-oxidant, anti-aging, anti-inflammatory, anti-nociceptive properties [15]. RF is found in most foods, with the highest content in dairy products, meat, and dark green vegetables [17,18]. Thus, due to the importance of this vitamin, a large number of data are found in the literature about its electrochemical determination [19,20,21,22,23].

Here, we report, for the first time, the electrochemical determination of RF with a novel 2D-MoS_2_-MoO_3_CC/SPCE sensor. To the best of our knowledge, there are no reports about the fabrication and application of a such SPCE electrochemical platform modified with a layer-by-layer structure based on molybdenum compounds grown/deposited on a carbon cloth network structure.

## 2. Materials and Methods

### 2.1. Preparation of the 2D-MoS_2_-MoO_3_CCcomposite

The 2D-MoS_2_-MoO_3_CC composite were fabricated as follows: first, pristine carbon cloth substrates were washed for 2 to 3 times with acetone and deionized water under sonication for 2 h, to remove organic residues and other impurities thoroughly. The cleaned carbon cloth substrates were kept in a hot air oven overnight. They are constituted of long nanofibers. Afterwards, the dry substrates were placed in a radio-frequency magnetron reactive sputtering (Huttenger, Germany) in a customized down setup sputtering mode and $Ar^{+}\;$(99.999%) is used as working gas and $O_{2}^{-}\;$(99.999%) as reactive gas in 1:5 ratio. The Mo metal target was fixed in the working pressure of 10^−2^ mbar for depositing a thin layer. A α-MoO_3_ thin film (around 350 nm thick) was deposited on carbon cloth substrates at deposition rate of 3 Ǻ/s at 450ºC temperature. The surface contamination on the target material was removed by pre-sputtering the target at 0.01 mbar pressure for 10 min. The RF power was set to 150W, the distance between target and substrate was maintained at 50 mm [9]. 2D-MoS_2_ nanosheets were prepared by liquid phase exfoliation (LPE) in sodium cholate (SC) watery solutions (1.5 mg/mL) [6]. Solutions were prepared by tip sonication (Branson S250) of MoS_2_ powder (particle size < 2µm, Sigma Aldrich) in SC at a concentration of 5 mg/mL for 30 min. Samples were kept in an ice bath to reduce detrimental heating effects during sonication. The dispersions were allowed to decant overnight in a flask. Then, the half top part was centrifuged at 1500 rpm for 90 min and the supernatant, rich of few-layer nanosheets, was collected. The dispersions thus obtained contained MoS_2_ flakes with an average number of layers, n = 9, an average lateral of 170 nm, in an estimated concentration of 80 µg/mL. The dispersions were stable for months [6]. Finally, the 2D-MoS_2_ suspension was drop casted onto the MoO_3_ carbon cloth and left to dry at room temperature.

### 2.2. Characterization

SEM images were acquired by means of a Zeiss CrossBeam 540 apparatus equipped with EDX detector. Raman spectroscopy was carried out with a XploRA micro-spectrometer (Horiba Scientific), with excitation at 638 nm. The laser beam was focused with a 100X objective (NA 0.9, WD 0.21 mm) on a diffraction limited spot. The Raman signal was collected through the same objective in a backscattering configuration and dispersed by an 1800 lines/mm grating onto a charge-coupled device (CCD) detector (Syncerity, Horiba Scientific). The laser power was set at 0.2 mW in order to avoid sample damaging and the signal was integrated over 30 s.

### 2.3. Modified Electrode Fabrication

To fabricate the modified SPCE, slices of pristine CC, MoO_3_-CC and MoS_2_-MoO_3_-CC were cut in order to cover entirely the surface of the working electrode of SPCE. Then, 6 µL of Nafion solution 5% was dropped on that for enhancing the adhesion on the surface of SPCE and left to dry at room temperature.

### 2.4. Electrochemical Measurements

Electrochemical analyses (Cyclic Voltammetry and Linear Sweep Voltammetry) were performed by using a DropSens µStat 400 Potentiostat empowered by Dropview 8400 software for data acquisition. The sensors were characterized by cyclic voltammetry (CV) and linear sweep voltammetry (LSV) in aerated 1M Phosphate-buffered saline (PBS) electrolyte. CV and LSV tests were carried out at a scan rate of 50 mV/s in the potential range from −0.8 to 0 V, by varying the concentration of the investigated analyte. The calibration curves were obtained by plotting the Faradaic current vs. analyte’s concentration. The sensitivity was computed as the slope of the calibration curve.

## 3. Results and Discussion

### 3.1. 2D-MoS_2_-MoO_3_-CC/SPCE Characterization

**Figure 1:**
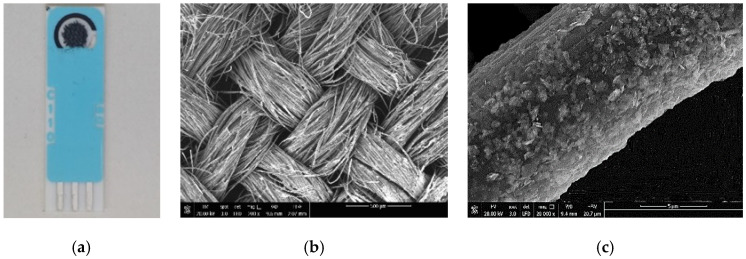
(**a**) Picture of the modified 2D-MoS_2_-MoO_3_-CC/screen-printed carbon electrode (SPCE); (**b**) SEM image of the 2D-MoS_2_- MoO_3_-CC network. (**c**) SEM image of the surface of a single 2D-MoS_2_-MoO_3_-CC/SPCE fiber.

A picture of the modified 2D-MoS_2_-MoO_3_-CC/SPCE is reported in Figure 1a. The sensitive area is 0.125 cm^2^. The network morphology of the 2D-MoS_2_-MoO_3_-CC fixed on the working electrode of SPCE is shown in Figure 1b. The large surface of carbon cloth fibers is beneficial for growing the MoO_3_ layer and successively to anchoring the 2D-MoS_2_ nanosheets (Figure 1c).

**Figure 2:**
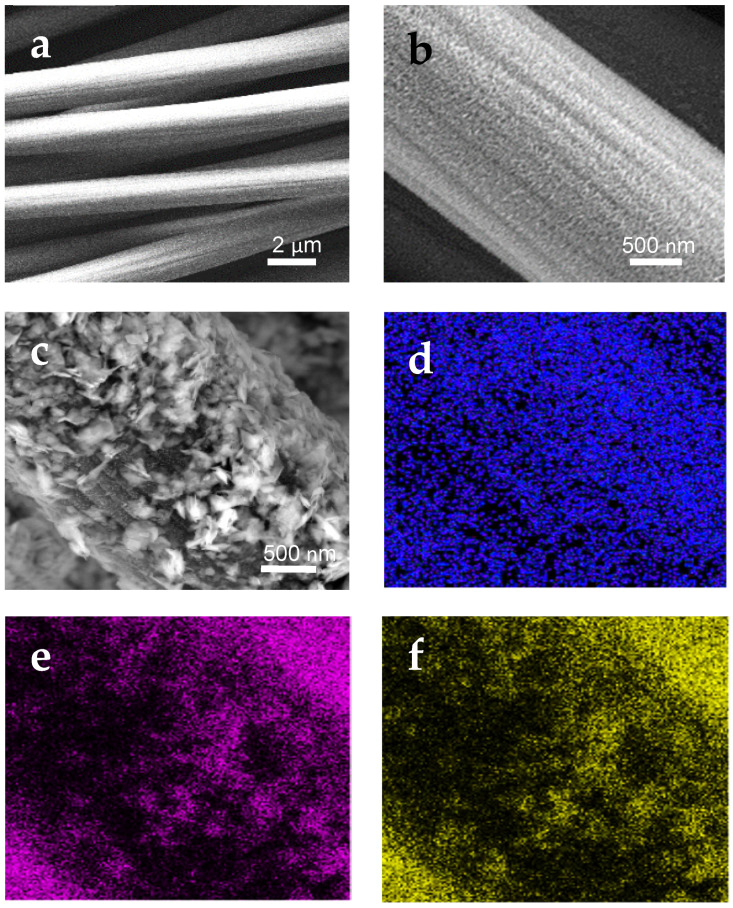
SEM images of: (**a**) CC/SPCE; (**b**) MoO_3_-CC/SPCE; (**c**) MoS_2_-MoO_3_-CC/SPCE. (**d**–**f**) energy dispersive X-ray (EDX) mapping of the SEM image shown in (**c**) at the O, Mo, and S energies, respectively.

This open network structure facilitates the electrolyte ions diffusion during the electrochemical tests. The change of morphology subsequent to the CC surface modification is shown in Figure 2.

Unmodified CC fibers present a smooth surface (Figure 2a). In the MoO_3_-CC, the MoO_3_ thin layer covers homogeneously the entire surface of the carbon fibers (Figure 2b). In Figure 2c is shown the two-layers 2D-MoS_2_-MoO_3_-CC composite. The MoS_2_ nanosheet particles are clearly seen on the top of underling MoO_3_ nanoparticle layer. There are no remarkable aggregation of MoS_2_ sheets on the MoO_3_ layer. Furthermore, an increase in the surface roughness is observed, which gives rise to higher active surface area for the modified electrode, and thus enhances its electrochemical activity. The 2D-MoS_2_ nanosheets build up an external porous layer covering the MoO_3_-CC fibers. EDX elemental mapping analysis (Figure 2d–f) of the MoS_2_-MoO_3_-CC composite-based SPCE confirms the presence and distribution of the main O, Mo, and S elements on the electrode surface.

**Figure 3:**
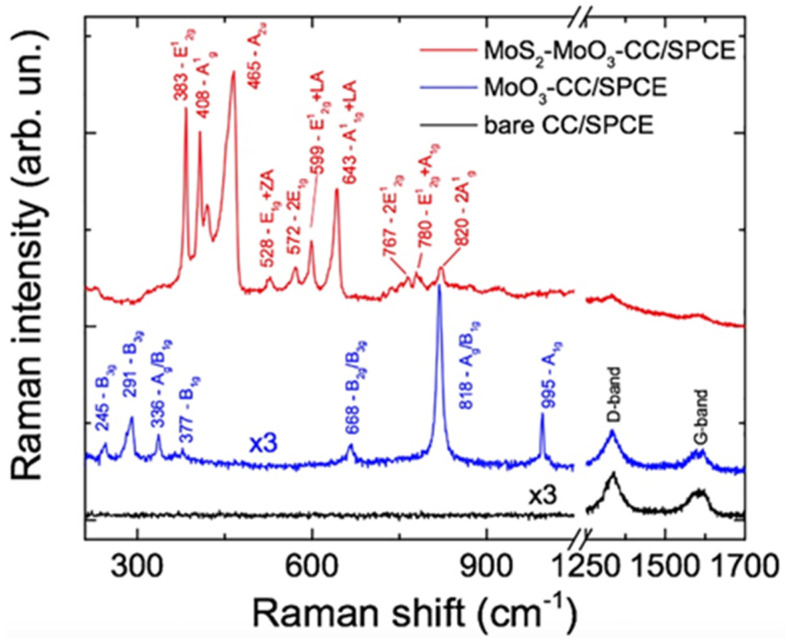
Raman spectra carried out at 638 nm on the CC/SPCE (black line), MoO_3_-CC/SPCE (blue line) and MoO_3_-CC-MoS_2_/SPCE (red line). The spectra on CC and MoO_3_-CC were multiplied by a factor 3 to fit the same intensity scale of the MoS_2_ one. All spectra were offset for clarity.

Raman spectra at 638 nm have been acquired first on the bare carbon cloth SPCE (Figure 3, black line), then on the carbon cloth added with molybdenum oxide SPCE (Figure 3, blue line) and finally on the MoS_2_-MoO_3_-CC/SPCE (Figure 3, red line). On the CC/SPCE, only the Raman fingerprint of carbon is detected, namely the D-band peak centered at 1330 cm^−1^ and the G-band around 1600 cm^−1^. These two peaks correspond to the amorphous carbon phase present in the carbon cloth [24].

On the MoO_3_CC/SPCE (blue line) we observe the superposition of the carbon bands and the MoO_3_ vibrational modes which are attributed to the α-MoO_3_ orthorhombic phase [25]. The spectrum of the MoS_2_-MoO_3_-CC/SPCE is dominated by the MoS_2_ signal. At 638 nm the photons are almost resonant with the B-excitonic transition of MoS_2_ [26], leading to resonant Raman (RR) effects [26,27]. When the frequency of the incoming light comes close to the specific frequency needed to drive the transfer of an electron from an occupied state to an unoccupied state, the absolute Raman intensity can change by several orders of magnitude. Besides the amplification of first-order transitions, RR scattering causes second-order Raman processes to be particularly amplified. These exhibit features coming from different crystalline momenta, potentially from the entire Brillouin zone (BZ), with the only constraint of negligible momentum of the phonon pairs involved in the two-phonon process. The strong second order transitions in MoS_2_ cover the 500–850 cm^−1^ frequency range. Table 1 summarizes the Raman modes observed on our sample, their symmetry and transition order. The position of the first-order transitions E^1^_2g_ (383 cm^−1^) and A^1^_g_ (408 cm^−1^) is typical of the 2H phase on few layers 2D-MoS_2_ [6,27]. The 466 cm^−1^ peakhas been attributed to either an A_2u_(Γ) vibration [28] or to an overtone of a longitudinal acoustic (LA) phonon [29].

### 3.2. Electrochemical Characterization

**Figure 4:**
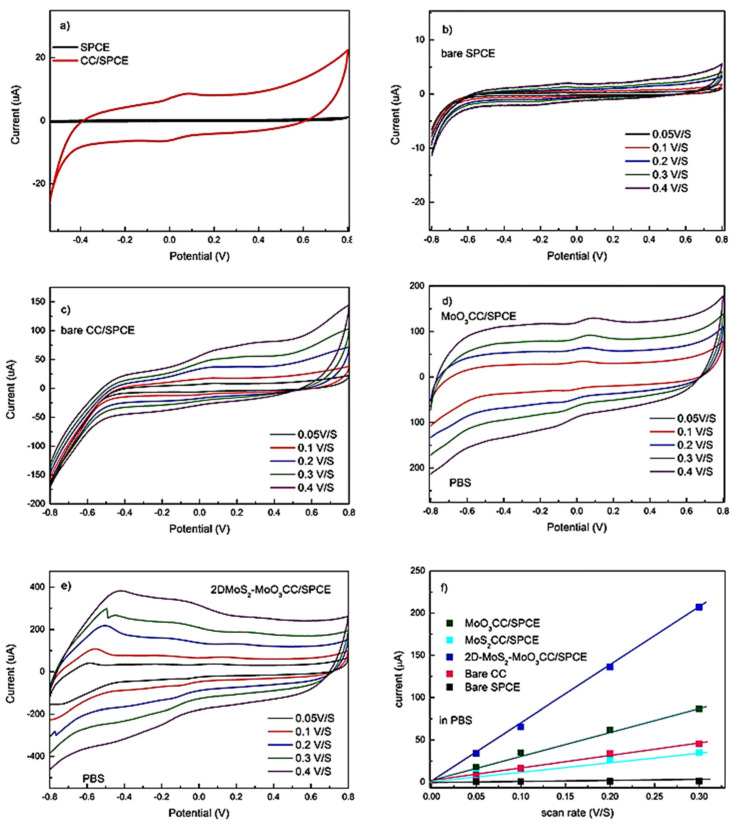
(**a**) Cyclic voltammetry (CV) spectra of the bare SPCE and modified carbon cloth (CC)/SPCE at 0.05 V/s in 1M PBS. CVs at different scan rates of: (**b**) bare SPCE, (**c**) bare CC/SPCE, (**d**) MoO_3_CC/SPCE and (**e**) 2D-MoS_2_-MoO3CC/SPCE from 0.05 V/s to 0.4 V/s. (**f**) Linear fitting of the capacitive currents of the different electrodes vs. scan rates.

A series of electrochemical tests has been carried out to characterize the bare screen-printed carbon electrodes and the modified ones. Preliminary CV tests have been conducted in 1 M PBS. Figure 4a compares the CV spectra of the bare SPCE and modified CC/SPCE at 0.05 V/s. The strong enhancement of the background current of the CC/SPCE, is due to its large electrochemical double-layer capacitance (EDLC). Cyclic voltammograms of the fabricated electrodes at different scan rates have been also collected. Each point related to sensor response reported is the average of three independent measurements. The standard deviation associated with these measurements has been evaluated to be less than 10%, which is good for these not yet optimized sensors.

As expected, the scan rate amplifies the capacitive current. This is well evident for the modified electrodes (compare Figure 4b–e), due to the exposure of more active sites on the working electrode’s surface. Plotting the scanning rate versus the current for all electrodes (Figure 4f), a series of straight lines were obtained, allowing us to estimate the EDLC for these electrodes from the slope and the geometrical area. These data show that the EDLC of the CC/SPCE is increased largely compared to the bare SPCE (black line). The EDLC is increased by nearly a factor 2 when the MoO_3_ layer is grown on CC/SPCE (dark green line) and undergoes a further strong enhancement when the MoS_2_ nanosheets are deposited (blue line). The 2D-MoS_2_-MoO_3_CC/SPCE composite electrode shows the highest EDLC (blue line). This suggests that the network structure of CC is able to provide a larger surface area compared to bare SPCE, which is further increased in the presence of the layered MoO_3_ and hybrid 2D-MoS_2_-MoO_3_ structure.

**Figure 5:**
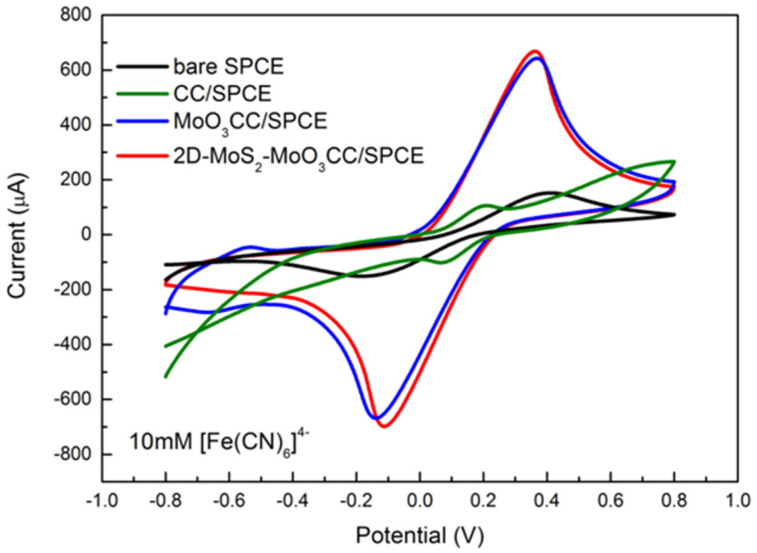
Cyclic voltammograms performed in 10 mM [Fe(CN)_6_]^4−^ at a scan rate of 0.05 V/swithbare SPCE (black line), CC/SPCE (green line),MoO_3_CC/SPCE (blue line), and 2D-MoS_2_-MoO_3_CC/SPCE (red line).

The electron transfer capability of the various electrodes has been tested with [Fe(CN)_6_]^4−^ as analyte (10 mM in 1 M PBS) by varying the scan rate from 0.05 to 0.4 V/s. In order to provide a quick comparison among the fabricated electrodes, Figure 5 displays the cyclic voltammograms obtained at a scan rate of 0.05 V/s.

As can be noted, both the current peak intensity (Ip) and the peak-to-peak separation (∆Ep) depend on the investigated electrodes. ∆Ep and Ip are helpful parameters to provide a qualitative estimation of the electron transfer rate due to the redox process at the electrode’s surface. In Table 2 we report the values of the anodic (Ipa) and cathodic (Ipc) current peaks, together with the value of ∆Ep for the bare SPCE, the modified CC/SPCE, the MoO_3_CC/SPCE, and 2D-MoS_2_-MoO_3_CC/SPCE measured with 10 mM [Fe(CN)_6_]^4−^ at a scan rate of 0.05 V/s.

**Figure 6:**
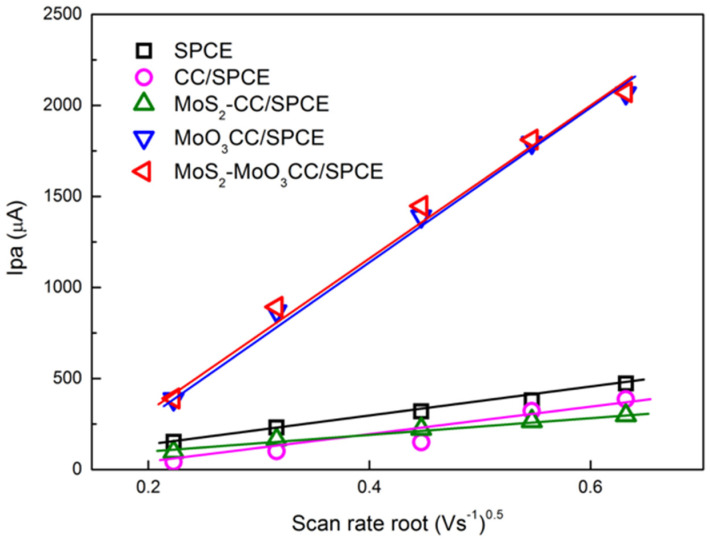
Plot of the anodic peak current (Ipa) vs. scan rate root. Ipa values were derived by CVs performed with the different bare and modified SPCE in 10 mM [Fe(CN)_6_]^4−^ at different scan rate.

Lower ∆Ep values and higher Ipa and Ipc is measured using the modified electrodes. These values suggest a faster electron transfer in the carbon cloth composite electrodes compared to bare SPCE likely resulting from a larger reaction surface area. Furthermore, the ΔEp values as well as the current peaks increase linearly with the square root of the scan rate (see Figure 6 for the anodic peak), indicating a diffusion-controlled process.

These studies suggest that the electrochemical behavior of the bare electrode is improved after modification with CC-based MoS_2_ and MoO_3_ layer. Further, it is evidenced that MoO_3_CC/SPCE and MoS_2_-MoO_3_CC/SPCE present almost similar electrochemical properties for the [Fe(CN)_6_]^4−^/[Fe(CN)_6_]^3−^ redox process, indicating that they have comparable EDLC and electron transfer capability.

### 3.3. Electrochemical Behavior in Presence of Riboflavin

We have exploit the enhanced electrochemical performances of the 2D-MoS_2_-MoO_3_ layer on CC to develop an electrochemical sensor for biomolecules detection. Here, we tested the new electrode on riboflavin (RF) at a concentration of 100 µM.

**Figure 7:**
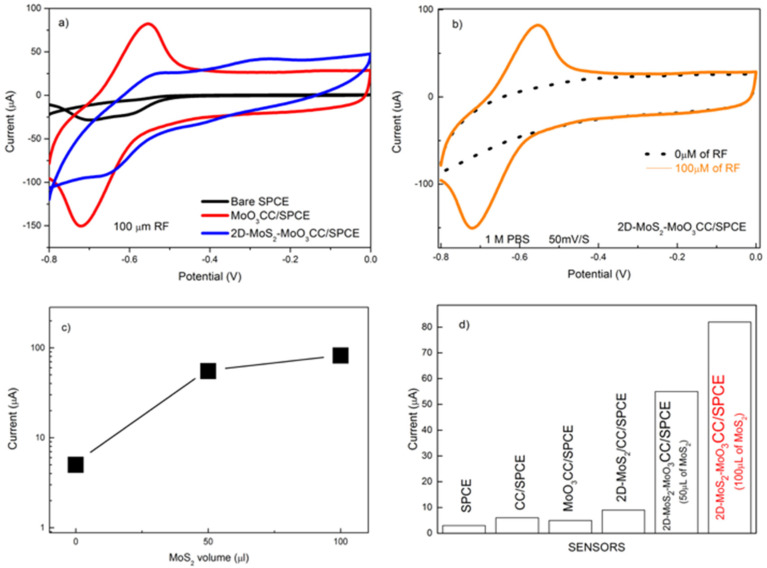
(**a**) CV of the bare SPCE (black line), MoO_3_CC/SPCE (blue line) and 2D-MoS_2_-MoO_3_CC/SPCE (red line) in presence of 100 µM RF; (**b**) CV of 2D-MoS_2_-MoO_3_CC/SPCE in absence (black dotted line) and in presence of 100 µM RF (orange line);(**c**) Ipa current vs. the volume of 2D-MoS_2_ nanosheets suspension used for the MoO_3_CC matrix modification; (**d**) comparison of the Ipa current obtained with the different electrodes in presence of 100µM riboflavin (RF).

Figure 7a shows the remarkable enhancement of the CV signal measured with the modified 2D-MoS_2_-MoO_3_CC/SPCE (red lines) compared to the bare SPCE (black lines) and MoO_3_CC/SPCE. This behavior highlight the strong effect of the MoS_2_ layer on RF electrocatalysis. Figure 7b presents the CV curves of the 2D-MoS_2_-MoO_3_CC/SPCE in absence (black dotted line) and in presence of RF (orange line). At the starting potential of −0.8 V, RF exists in its reduced form. At −0.55V, RF exhibits an oxidation peak followed by a reduction peak at −0.72V on the back forward scan. This electrochemical redox process involves the transfer of two protons and two electrons [29]. We have subsequently checked the effect of the loading of MoS_2_ on the MoO_3_CC fiber network. As shown in Figure 7c, the current increases as a function of the MoS_2_ quantity on the MoO_3_CC matrix, thus proving the fundamental electrocatalytic role of MoS_2_ in enhancing the response to RF.

Figure 7d compares the current response of the different electrodes in the determination of RF. The sensor employing the modified 2D-MoS_2_-MoO_3_CC electrode has the highest sensitivity towards riboflavin, dwarfing the performances of other electrodes. The better sensitivity of the composite electrode can be associated to the increase of the effective surface area of the working electrode and the formation of new electroactive sites, formed at the interface between the MoO_3_ and 2D-MoS_2_ layers.

**Figure 8:**
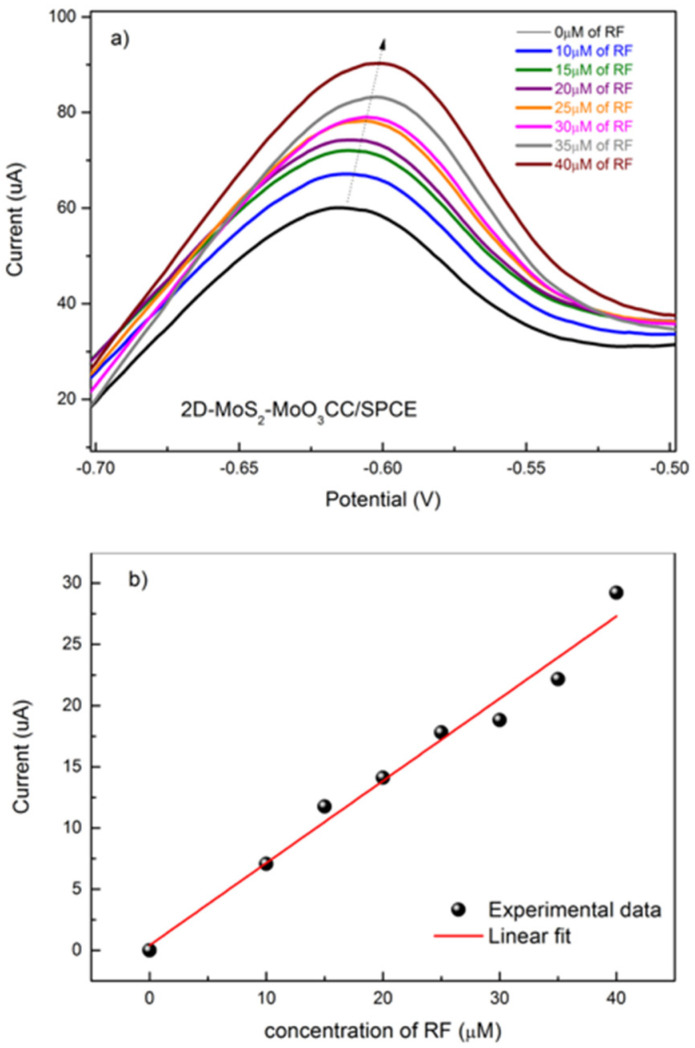
(**a**) Linear sweep voltammetry (LSV) of 2D-MoS_2_-MoO3CC/SPCE, performed in 1 M PBS electrolyte and in the presence of different concentrations of RF. (**b**) Calibration curve for the determination of RF.

Based on these results, we have evaluated the response of the 2D-MoS_2_-MoO_3_CC-based sensor at different RF concentrations. In Figure 8a, is reported the linear sweep voltammetry (LSV) analysis of solutions containing increasing concentrations of RF, from 0 to 40 µM, evidencing the associated augmentation of the peak current value. In Figure 8b (black dots), is shown the calibration curves for RF, plotting the peak current as a function of the analyte concentration. The sensitivity, namely the slope of the calibration curve, is computed from a linear fit of the data (red line), and is 0.67 µA µM^−1^. LOD was 1.5 µM, as calculated by comparing the signals (S) from the samples with known and low concentrations of RF with those of blank samples (N) and by establishing the minimum concentration at which the RF signal is three times as high as noise (S/N = 3).

The performances (linear range, sensitivity and limit of detection) of the proposed 2D-MoS_2_-MoO_3_CC/SPCE based sensor have been compared to most of the recently reported riboflavin electrochemical sensors (see Table 3).

**Figure 9:**
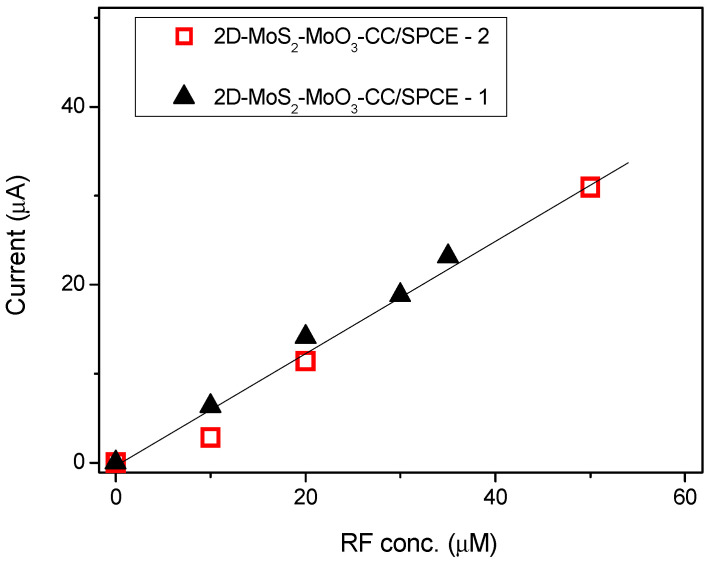
Responses versus riboflavin concentrations with two different 2D-MoS_2_-MoO_3_CC/SPCE sensors. Sensors 2 was fabricated about one year after Sensor 1, using newly prepared 2D-MoS_2_ nanosheets.

From this comparison it can be deduced that our sensor platform displays a wide linear range and a high sensitivity respect to the other sensors. We repeated this test after about one year using a new prepared 2D-MoS_2_ nanosheets suspension for replicating the fabrication of a new 2D-MoS_2_-MoO_3_CC/SPCE sensor in the same conditions of the first one. The calibration curves obtained by these tests are compared in Figure 9. It can be clearly observed that the two set of data points can be fitted almost well from the same linear relationship. The reported findings suggest that the electrochemical properties of the 2D-MoS_2_-MoO_3_CC network structure as well as the fabrication procedure of 2D-MoS_2_-MoO_3_CC/SPCE platform can be replicated very well, leading to different sensor devices with reproducible response.

The effect of some interferent biomolecules has been also investigated. Folic acid (FA), which is another vitamin of B group, and ascorbic acid (AA), a vitamin of C group, are considered as the main interferent analytes in the determination of RF. These two biomolecules are present in human body so it’s mandatory to verify if their presence affect the RF detection. Preliminary tests have shown that these compounds, in absence of RF, show no redox peak in the potential range where the RF peak is present. This is an expected result because, similarly to redox processes of most organic compounds, they take place in the positive potential range.

**Figure 10:**
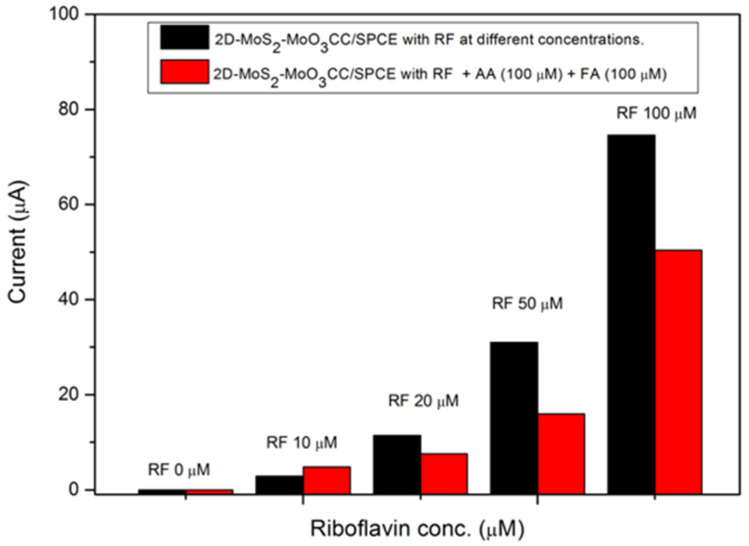
Ipa current values registered by the 2D-MoS_2_-MoO_3_CC/SPCE sensor in presence of RF at different concentrations (black bars) and in co-presence (red bars) of FA and AA at 100 µM in 1 M PBS.

The effect of FA and AA on the determination of riboflavin has been investigated with our 2D-MoS_2_-MoO_3_CC/SPCE sensor. The test has been carried out at different concentrations of RF in presence of the two analytes. LSV curves measured in a solution of RF mixed with 100µM AA and 100µM FA are reported in Figure 10. The test shows clearly how the presence of the two interferent analytes decreases the RF current peak. This behavior can be explained assuming that FA and AA compete with RF for the interaction with a significant fraction of the active site on the sensing layer.

The above results indicate that the effects of interferents substances on the RF sensor response needs to be checked. As it is well known, the electrochemical characteristics of the biomolecules here investigated are largely dependent on pH [35,36,37]. This implies that it is possible to optimize the conditions at which riboflavin has the larger interaction with the electrode surface, which means that the sensor response will be probably less influenced by the presence of these interferent analytes. This is a general strategy which can be used for monitoring riboflavin in real samples [38,39], thus we have planned to investigate in detail this aspect aim at applying our developed sensor in real applications.

## 4. Conclusions

A 2D-MoS_2_-MoO_3_CC nanocomposite electrode was prepared and tested for the electrochemical detection of riboflavin in PBS. The modified platform was fabricated by layers of MoO_3_ and 2D-MoS_2_ nanosheets on the fiber of CC network structure. The MoO_3_ layer was grown on CC fibers through radio-frequency magnetron reactive sputtering. An additional layer of 2D-MoS_2_ nanosheets was casted, starting from a solution of MoS_2_ few-layers flakes produced by LPE.

The structure was used for fabricating a novel electrochemical platform with enhanced properties with respect to the conventional commercial devices and those prepared with the single constituents. Riboflavin electroxidation was observed at negative potential (around −0.55 V) and this can be regarded as an advantage, because most of the redox processes of organic compounds which can interfere with it take place at more positive potentials. Interference from folic and ascorbic acid has been observed to decrease the sensitivity of the sensor for riboflavin, although not dramatically.

The enhanced sensitivity of the 2D-MoS_2_-MoO_3_CC/SPCE sensor was attributed to the improved electron transport provided by the conductive CC structure network, as well as the enhancement of specific active sites for the electrocatalytic reduction/oxidation of RF on MoS_2_. This unique 2D-MoS_2_-MoO_3_CC/SPCE electrochemical platform resulted in a promising sensor device for the electroanalytical determination of riboflavin.

## Figures and Tables

**Table 1:** Peaks frequency, group theory representation and attribution of the modes observed in the Raman spectra of the different samples [24,25,28,29].

Sample	Energy (cm^−1^)	Symmetry	Attribution
MoO_3_	245	B_3g_	τ O=Mo=O
MoO_3_	291	B_3g_	ω O=Mo=O
MoO_3_	336	A_g_/B_1g_	δ O-Mo-O
MoO_3_	377	B_1g_	δ O=Mo=O (scissoring)
MoO_3_	668	B_2g_/B_3g_	ν O–Mo–O
MoO_3_	818	A_g_/B_1g_	ν_s_ O=Mo=O
MoO_3_	995	A_g_	ν_as_O=Mo=O
CC	1330	A_1g_	D-band
CC	1605	E_2g_	G-band
MoS_2_	383	E^1^_2g_(Γ)	First-order
MoS_2_	408	A^1^_g_(Γ)	First-order
MoS_2_	420	B’	First-order
MoS_2_	465	A_2u_(Γ) or 2LA(M)	First or second order
MoS_2_	528	E_2u_(M) + LA(M)	Second-order
MoS_2_	572	2E_1g_(Γ)	Second-order
MoS_2_	599	E^1^_2g_(Γ) + LA(M)	Second-order
MoS_2_	643	A^1^_1g_(M) + LA(M)	Second-order
MoS_2_	767	2E^1^_2g_(Γ)	Second-order
MoS_2_	780	E^1^_2g_ + A^1^_g_	Second-order
MoS_2_	820	2 A^1^_g_(Γ)	Second-order

**Table 2:** CV data of anodic (Ipa) and cathodic (Ipc) current peaks and ∆Ep for the bare SPCE and the modified CC/SPCE, MoO_3_CC/SPCE, and 2D-MoS_2_-MoO_3_CC/SPCE in 10 mM [Fe(CN)_6_]^4–^ at a scan rate of 0.05 V/s.

Electrode	Ipa (µA)	Ipc (µA)	ΔEp (V)
SPCE	150	−150	0.57
CC/SPCE	110	−107	0.13
MoO_3_-CC/SPCE	642	−664	0.46
MoS_2_-MoO_3_/SPCE	671	−693	0.495

**Table 3:** Comparison of MoS_2_-MoO_3_CC/SPCE sensor performance with recently reported riboflavin electrochemical sensors.

Electrode Modifier	Method	Linear Range	Sensitivity *	LOD	Ref.
Cr-SnO_2_	DPV	0.2 nM–0.1 mM	0.047	0.11 nM	[20]
ssDNA-MoS_2_-Gr **	DPV	25 nM–2.25 mM	0.83	20 nM	[30]
CC ***	AMP	5 nM–100 nM	**-**	2.2 nM	[31]
MnO_2_	DPV	20 nM–9 μM	NA	15 nM	[32]
Co^2+^-Y zeolite	CV	1.7–3.4 μM	NA	0.71 μM	[33]
MnO_2_	DPV	2 μM–0.11 mM	NA	15 nM	[34]
MoS_2_-MoO_3_CC	LSV	2 μM–40 μM	0.67	1.5 μM	This work

* Sensitivity is here defined as µA µM^−1^. ** Gr = graphene. *** CC = carbon cloth-RF-cytochrome C.
